# Genotyping of a tri-allelic polymorphism by a novel melting curve assay in *MTHFD1L*: an association study of nonsyndromic Cleft in Ireland

**DOI:** 10.1186/1471-2350-13-29

**Published:** 2012-04-20

**Authors:** Stefano Minguzzi, Anne M Molloy, Kirke Peadar, James Mills, John M Scott, James Troendle, Faith Pangilinan, Lawrence Brody, Anne Parle-McDermott

**Affiliations:** 1 Nutritional Genomics Group, School of Biotechnology, Dublin City University, Dublin, Ireland; 2 School of Medicine, Trinity College Dublin, Dublin 2, Ireland; 3 Child Health Epidemiology Unit, Health Research Board, Dublin, Ireland; 4 Department of Health and Human Services, Eunice Kennedy Shriver National Institute of Health, Bethesda, MD, USA; 5 School of Immunology & Biochemistry, Trinity College Dublin, Dublin 2, Ireland; 6 Molecular Pathogenesis Section, Genome Technology Branch, National Human Genome Research Institute, Bethesda, MD, USA

## Abstract

**Background:**

Polymorphisms within the *MTHFD1L* gene were previously associated with risk of neural tube defects in Ireland. We sought to test the most significant *MTHFD1L* polymorphisms for an association with risk of cleft in an Irish cohort. This required the development of a new melting curve assay to genotype the technically challenging *MTHFD1L* triallelic deletion/insertion polymorphism (rs3832406).

**Methods:**

Melting curve analysis was used to genotype the *MTHFD1L* triallelic deletion/insertion polymorphism (rs3832406) and a Single Nucleotide Polymorphism rs17080476 in an Irish cohort consisting of 981 Irish case-parent trios and 1,008 controls. Tests for association with nonsyndromic cleft lip with or without cleft palate and cleft palate included case/control analysis, mother/control analysis and Transmission Disequilibrium Tests of case-parent trios.

**Results:**

A successful melting curve genotyping assay was developed for the deletion/insertion polymorphism (rs3832406). The TDT analysis initially showed that the rs3832406 polymorphism was associated with isolated cleft lip with or without cleft palate. However, corrected p-values indicated that this association was not significant.

**Conclusions:**

Melting Curve Analysis can be employed to successfully genotype challenging polymorphisms such as the *MTHFD1L* triallelic deletion/insertion polymorphism (DIP) reported here (rs3832406) and is a viable alternative to capillary electrophoresis. Corrected p-values indicate no association between *MTHFD1L* and risk of cleft in an Irish cohort.

## Background

Cleft lip with or without cleft palate (CLP) and cleft palate only (CPO) are common birth defects of complex and heterogeneous aetiology. Previous studies suggest that folate deficiency before or during pregnancy can increase risk of clefting in the resulting offspring [1-4]. Folate supplementation in pregnancy has been shown to reduce the recurrence of CLP in families and to have a modest reduction in birth prevalence on a population basis [5]. Nevertheless this association is still controversial [6,7]. Numerous candidate gene association studies between clefts and folate related genes have shown mixed results and include methylenetetrahydrofolate reductase (*MTHFR* [Genbank: NP_005948.3]) [4,8-16], methylenetetrahydrofolate dehydrogenase (NADP + dependent) (*MTHFD1* [Genbank: NP_005947.3]) [1,16,17], 5,10-methenyltetrahydrofolate synthetase (*MTHFS* [Genbank: NP_001186689.1]) and methionine synthase (*MTR* [Genbank: NP_000245.2]) [4,17-19]. However, candidate gene studies to date have not considered *MTHFD1L* [Genbank: NP_001229696.1] in relation to nonsyndromic clefts. Environmental factors were reported for this cohort previously [16] and included data on the mother’s medication use, folic acid exposure, alcohol and smoking. No interaction between genotype and these environmental factors were found in that study.

Based on its association with neural tube defects (NTDs) [20], and the previously detected association of its cytoplasmic homologue MTHFD1 in our cleft cohort, we considered the mitochondrial enzyme MTHFD1L to be a prime candidate for consideration for association with cleft. The relevance of this gene is increasing given its identification in genome wide association screens as being associated with coronary artery disease [21,22] and Alzheimer’s disease [23]. Moreover a previous study has shown that MTHFD1L is upregulated in human colon adenocarcinoma [24]. The *MTHFD1L* gene encodes the mitochondrial C1-Tetrahydrofolate(THF) Synthase protein which has a monofunctional 10-formyl-THF synthetase activity while lacking the 5,10-methylene-THF dehydrogenase and 5,10-methenyl-THF cyclohydrolase activities typically found in the trifunctional cytoplasmic protein encoded by MTHFD1 [25]. It has been shown that the *MTHFD1L* gene produces 2 alternatively spliced mRNAs with the shorter transcript lacking synthetase activity [26]. Previously, we reported that the *MTHFD1L* rs3832406 DIP and numerous SNPs in linkage disequilibrium (LD) are associated with the risk of NTDs in the Irish population [20]. We proposed that the DIP polymorphism is the direct disease causing variant within the associated LD block by affecting alternative splicing of the gene [20].

In this study, we genotyped the *MTHFD1L* DIP rs3832406 and the most statistically significant NTD-associated SNP in the adjacent LD block i.e., rs17080476, in 981 Irish case-parent trios affected by CPL or CPO. We developed a melting curve method capable of genotyping deletion/insertion polymorphisms without the need for capillary electrophoresis.

## Methods

### Subjects

Buccal swab or blood samples were obtained at the Dublin Cleft Centre in Ireland as previously described [16] from subjects with cleft palate only (CPO) or cleft lip with or without cleft palate (CLP) along with their mothers and fathers. A total of 2,688 samples including 758 complete triads and 223 incomplete triads were collected for this study. Out of the total number of cleft cases this included 347 (33.8%) isolated CPO cases plus an additional 108 (10.5%) with multiple defects and 531 (51.7%) isolated CLP cases plus an additional 42 (4%) with multiple defects. All the cases of this study were non-syndromic. Multiple cases included children with one or multiple defects along with cleft. Chromosomal anomalies and other conditions (i.e. mother had diabetes or epilepsy or was exposed to potentially teratogenic drugs) were excluded. Control samples (n = 1,008) were collected from a population of 56,049 pregnant women attending the three main maternity hospitals in the Dublin area between 1986 and 1990 as previously described [16,27]. Written informed consent was obtained from all participants. Ethical approval was granted by the Research Ethics Committees of the Health Research Board of Ireland, the participating hospitals, and the Institutional Review Board at NIH.

### Genotyping

Genomic DNA was extracted from blood or buccal swab collected samples using a QIAamp DNA Blood Mini Kit (Qiagen, UK). HybProbe melting curve assays were designed to genotype DIP rs3832406 and SNP rs17080476 on a LightCycler 480 Real Time PCR machine (Roche) and are described in more detail below. Genotyping quality was verified by repeat genotyping of at least 10% of samples with agreement rate of ≫99% and overall success rate of ≫99%. In addition, 10% of the controls were genotyped by the HybProbe melting curve assays described here and compared to the assays used previously [20]. Comparison of control genotype calls gave a 95.7% agreement for DIP rs3832406 and 99% agreement for SNP rs17080476. All discrepant genotype calls for any sample were resolved by re-genotyping or were left out of the final analysis.

### SNP rs17080476 assay

SNP rs17080476 reagents and analysis conditions are: Forward Primer 5′-GCAACTTTGTTTAGTATGAAAATTTGAT-3′ (4 μM), reverse primer5′-TCTGTCTTCACCCAGCC (2 μM), anchor probe 5′-Bodipy630/650-AAGAGGGGAAAAAAAACCTTTCTCCATTATTCCTA-PHO-3′(0.4 μM), sensor probe 5′-ATTCATTTCTTTACAGCAGTGGGATTATGAAA-Fluorescein 3′ (0.2 μM), pre-incubation 10 minutes at 95°C, amplification 45 cycles of 15 seconds at 95°C, 15 seconds at 56°C, 15 seconds at 72°C, melting curve 1 minute at 95°C, 2 minutes at 50°C, acquisition ramp up to 80°C (0.11°C/s, 5 acquisitions per°C).

### DIP rs3832406 assay

DIP rs3832406 reagents and analysis conditions are: forward primer 5′-AAGCTTCCTGTTACCAC-3′ (4 μM), reverse primer 5′-AGGAGAATCACTTCAACC-3′ (2 μM), anchor probe: 5′-AGCCCCACGTTTGAATTTTATGTTTTTCCTAAAGT-Fluorescein-3′ (0.2 μM). Sensor probe: 5′BODIPY630/650-AGGGAAGATTATTATTATTATTATTATTATTATTTTCTTTTTCAGACGGA-Phosphate-3′ (0.2 μM),pre-incubation 10 minutes at 95°C, amplification 45 Cycles of 10 seconds at 95°C, 10 seconds at 56°C, 10 seconds at 72°C, melting curve 10 seconds at 95°C, 1 minute at 50°C, acquisition ramp up to 70°C (0.02°C/s, 30 acquisitions per°C).

### Statistical methods

Power calculations to detect an odds ratio of 1.5 assuming a dominant model for the case–control analyses were as follows: rs3832406 Allele 1 60%, Allele 2 95%, Allele 3 93%; rs17080476 G 95%. Assuming a recessive model: rs3832406 Allele 1 96%, Allele 2 36%, Allele 3 23%; rs17080476 G 34%. Our primary analysis was carried out with isolated nonsyndromic cases of CLP and CPO and their parents. A secondary analysis was then carried out including nonsyndromic cleft cases with other defects. Hardy-Weinberg equilibrium (HWE) was tested within each subject class (case, mother, father and controls) for each polymorphism by chi-squared test. Associations with CLP and CPO were tested for each polymorphism in cases/controls and separately in mothers/controls by logistic regression and odds ratios using either a dominant or recessive genetic disease model. Triads (case, mother, and father) were used to perform the Transmission Disequilibrium Test (TDT) of Spielman *et al*. [28]. The TDT P-values were adjusted using permutational correction [29].

## Results and discussion

### Development of a novel assay to genotype DIP rs3832406 by Melting Curve Analysis

**Figure 1:**
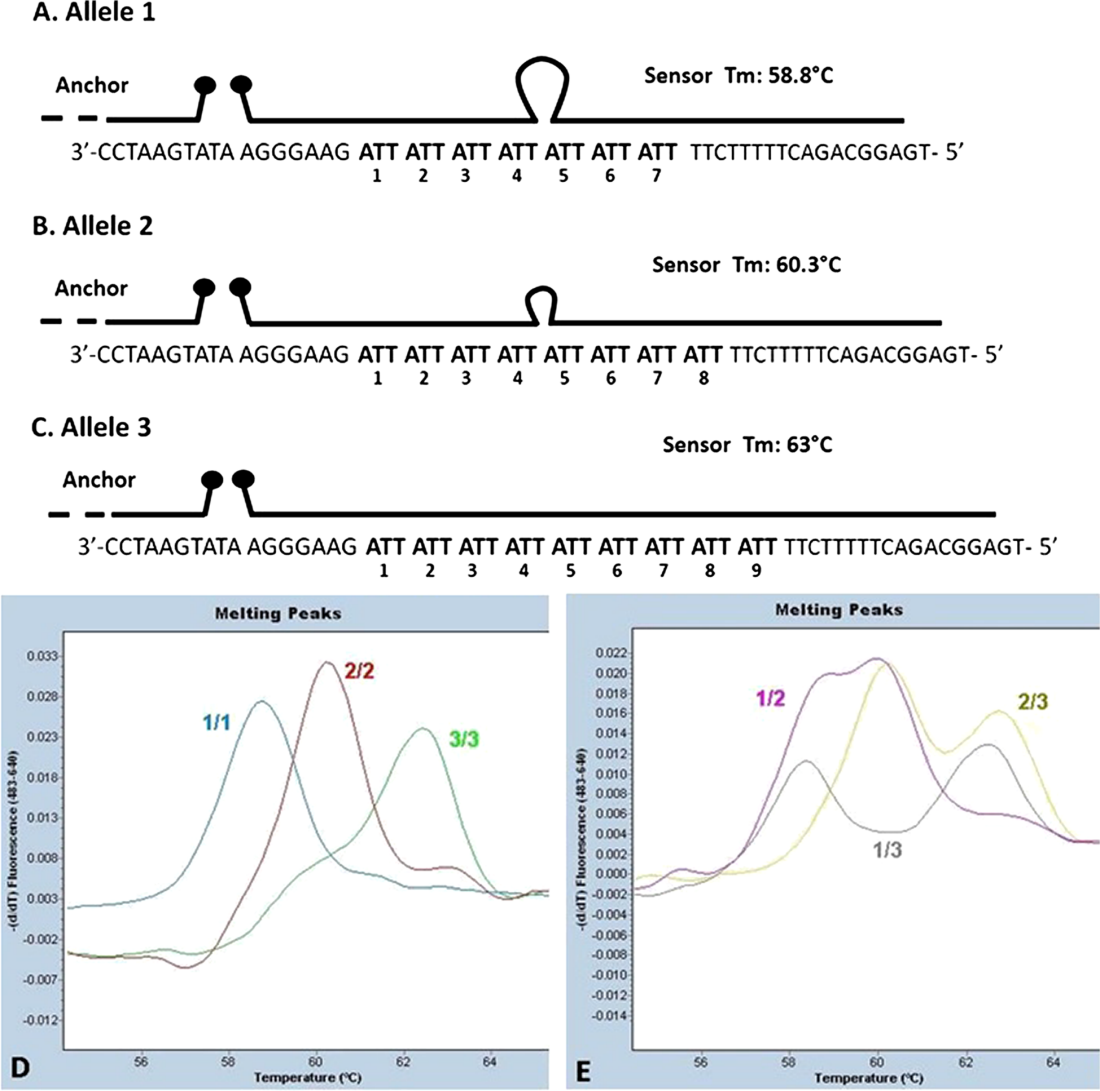
**Modified Melting Curve Analysis for DIP rs3832406.** The Sensor probe design for detection of all three alleles of DIP rs3832406 is shown. The Sensor probe is designed to perfectly match the complement of Allele 3. A. Sensor probe bound to Allele 1 has a Tm of 58.8°C. B. Sensor probe bound to Allele 2 has a Tm of 60.3°C. C. Sensor probe bound to Allele 3 has a Tm of 63°C. D. Examples of homozygote melting peaks for each of the three alleles. E. Examples of heterozygote melting peaks for all three allele combinations.

The *MTHFD1L* gene has received particular attention in recent years owing to its association with coronary artery disease, Alzheimer’s disease and NTDs. Our previous study, demonstrated that the *MTHFD1L* rs3832406 DIP is functional by impacting on alternative splicing efficiency [20]. We report a new modified melting curve assay to genotype this functionally relevant triallelic *MTHFD1L* polymorphism without the need for traditional capillary electrophoresis methods. A single assay which is able to distinguish 3 alleles contemporaneously was developed taking advantage of the GC-rich regions flanking the DIP (Figure 1). As described previously [20], this polymorphism is a repeated “ATT” sequence that has three common alleles, Allele 1 (ATT_7_) Allele2 (ATT_8_) and Allele 3 (ATT_9_). A wide 50-base sensor probe was designed to perfectly match Allele 3 with 9 ATT repeats and its flanking regions, producing a melting temperature (Tm) of 63°C (Figure 1c). The same probe produces a 3-base mismatched bubble on Allele 2 and a 6-base mismatched bubble on Allele 1 causing a Tm of 60.3°C and 58.8°C respectively (Figure 1a-b). The probe pairing starts from the GC-rich external regions allowing the formation of an internal mismatched bubble for Alleles 1 and 2. A slow acquisition ramp allowed melting peaks for each homozygote and heterozygote genotype to be distinguished (Figure 1d-e).

### DIP rs3832406, SNP rs17080476 and risk of CLP

We genotyped rs3832406 DIP and SNP rs17080476 in an Irish cleft cohort in a bid to test for association. The genotype frequencies of SNP rs17080476 and DIP rs3832406 in our CLP, CPO and control samples are shown in Table 1. Genotype distributions in all groups were in HWE. DIP rs3832406 showed an association with CLP case status based on TDT analysis (Table 2). The TDT analysis showed that Allele 1 is transmitted to the offspring 55.2% of times (p = 0.037) in isolated CLP cases, indicating that this allele is associated with increased disease risk. The addition of multiple case families to this analysis enhances the statistical significance (56.1% transmission, p = 0.011). Allele 3 has the lowest frequency and was passed to the offspring only 42.8% of times (p = 0.035) in multiple CLP cases, appearing to have a protective role against the disease. However, correction of these significant p-values using permutational adjustment resulted in loss of statistical significance. We did not observe statistical significance with SNP rs17080476 which shares a D’ value of 0.61 with DIP rs3832406 and represented the most statistically compelling variant from this genomic region in our NTD study [20] (Table 2). The majority of other analyses performed showed no significant association with the risk of cleft (Table 3).

**Table 1:** ***MTHFD1L*Genotyping Results in Triads (Cases, Mother and Fathers) and Controls for CLP and CPO (Isolated) or with Other Defects (Multiple)**

**DIPrs3832406**	Isolated defects						Multiple defects							
**CLP**	Fathers		Mothers		Cases		Fathers		Mothers		Cases		Controls	
	*n*	%	*n*	%	*n*	%	*n*	%	*n*	%	*n*	%	*n*	%
11	162	41.8	187	40.1	209	41.9	174	42.0	203	40.8	231	43.5	419	42.1
12	102	26.3	128	27.5	149	29.9	116	28.0	140	28.1	163	30.7	267	26.8
13	76	19.6	89	19.1	84	16.8	82	19.8	97	19.5	91	17.1	196	19.7
22	18	4.6	24	5.2	18	3.6	18	4.3	24	4.8	18	3.4	40	4.0
23	24	6.2	31	6.7	28	5.6	24	5.8	34	6.8	28	5.3	52	5.2
33	6	1.5	7	1.5	11	2.2	6	1.4	7	1.4	11	2.1	21	2.1
Total	388		466		499		420		505		542		995	
**CPO**	Fathers		Mothers		Cases		Fathers		Mothers		Cases			
	*n*	%	*n*	%	*n*	%	*n*	%	*n*	%	*n*	%		
11	98	37.3	118	38.1	134	41.7	145	40.8	154	36.9	175	41.0		
12	78	29.7	82	26.5	88	27.4	97	27.3	110	26.4	118	27.6		
13	60	22.8	69	22.3	62	19.3	77	21.7	99	23.7	86	20.1		
22	8	3.0	12	3.9	7	2.2	8	2.3	18	4.3	9	2.1		
23	18	6.8	20	6.5	23	7.2	25	7.0	26	6.2	29	6.8		
33	1	0.4	9	2.9	7	2.2	3	0.8	10	2.4	10	2.3		
Total	263		310		321		355		417		427			
**SNPrs17080476**	Isolated defects						Multiple defects							
**CLP**	Fathers		Mothers		Cases		Fathers		Mothers		Cases		Controls	
	*n*	%	*n*	%	*n*	%	*n*	%	*n*	%	*n*	%	*n*	%
AA	268	68.2	296	63.1	320	63.1	285	67.1	320	62.9	344	62.5	660	65.9
AG	110	28.0	160	34.1	170	33.5	124	29.2	176	34.6	188	34.2	308	30.8
GG	15	3.8	13	2.8	17	3.4	16	3.8	13	2.6	18	3.3	33	3.3
Total	393		469		507		425		509		550		1001	
	Isolated defects						Multiple defects							
**CPO**	Fathers		Mothers		Cases		Fathers		Mothers		Cases			
	*n*	%	*n*	%	*n*	%	*n*	%	*n*	%	*n*	%		
AA	173	65.5	205	65.7	214	65.6	234	65.9	274	65.6	288	66.8		
AG	86	32.6	93	29.8	100	30.7	115	32.4	127	30.4	125	29.0		
GG	5	1.9	14	4.5	12	3.7	6	1.7	17	4.1	18	4.2		
Total	264		312		326		355		418		431			

**Table 2:** TDT analysis for DIP rs3832406 and SNP rs17080476 in all cleft sample

		Allele	Passed		Not Passed		GRR^1^ (95% CI)	P-value
**DIP rs3832406**			*n*	%	*n*	%		
	**Isolated CLP**	1	194	55.6	155	44.4	1.3 (1.0, 1.5)	**0.0372**
		2	119	47.2	133	52.8	0.9 (0.7, 1.1)	0.3781
		3	88	43.8	113	56.2	0.8 (0.6, 1.0)	0.0786
		Total	401		401			
	**Multiple CLP**	1	216	56.5	166	43.5	1.3 (1.1, 1.6)	**0.0107**
		2	128	46.5	147	53.5	0.9 (0.7, 1.1)	0.2523
		3	92	42.8	123	57.2	0.7 (0.6,1.0)	**0.0351**
		Total	436		436			
	**Isolated CPO**	1	145	52.5	131	47.5	1.1 (0.9, 1.4)	0.3996
		2	86	47.8	94	52.2	0.9 (0.7, 1.2)	0.5511
		3	77	48.1	83	51.9	0.9 (0.7, 1.3)	0.6353
		Total	308		308			
	**Multiple CPO**	1	188	52.2	172	47.8	1.1 (0.9, 1.3)	0.3992
		2	115	48.9	120	51.1	1.0 (0.7, 1.2)	0.7443
		3	101	47.4	112	52.6	0.9 (0.7, 1.2)	0.4512
		Total	404		404			
**SNP rs17080476**	**Isolated CLP**	G	132	54.3	111	45.7	1.2 (0.9, 1.5)	0.1785
		A	111	45.7	132	54.3		
		Total	243		243			
	**Multiple CLP**	G	144	54.3	121	45.7	1.2 (0.9, 1.5)	0.1582
		A	121	45.7	144	54.3		
		Total	265		265			
	**Isolated CPO**	G	87	50.6	85	49.4	1.0 (0.8, 1.4)	0.8788
		A	85	49.4	87	50.6		
		Total	172		172			
	**Multiple CPO**	G	119	50.9	115	49.1	1.0 (0.8, 1.3)	0.7937
		A	115	49.1	119	50.9		
		Total	234		234			

^1^ GRR = genotype relative risk

^2^ CI = confidence interval

^3^ Significant values are marked in bold.

**Table 3:** Logistic regression analysis of case/controls and mother/controls for DIP rs3832406 and SNP rs17080476 in all cleft samples

	**Polymorphism/Allele**	**Name**	**Dominant**		**Recessive**		**Multiplicative**	
			OR^1^(95% CI^2^)	p- value	OR(95% CI)	p-value	OR (95% CI)	p-value
**Isolated CLP**	DIP Allele 1	Case-CTRL	1.1 (0.8, 1.5)	0.6159	1 (0.8, 1.3)	0.8468	1 (0.9, 1.2)	0.7022
		Mother-CTRL	0.9 (0.6, 1.2)	0.3917	0.9 (0.7, 1.1)	0.4774	0.9 (0.8, 1.1)	0.3476
	DIP Allele 2	Case-CTRL	1.1 (0.9, 1.4)	0.3358	0.8 (0.5, 1.4)	0.4926	1.1 (0.9, 1.3)	0.5534
		Mother-CTRL	1.1 (0.9, 1.4)	0.2363	1.2 (0.7, 2)	0.5077	1.1 (0.9, 1.4)	0.2176
	DIP Allele 3	Case-CTRL	0.9 (0.7, 1.1)	0.1928	1 (0.5, 2)	0.9158	0.9 (0.7, 1.1)	0.2349
		Mother-CTRL	1 (0.8, 1.3)	0.9044	0.7 (0.3, 1.5)	0.3311	1 (0.8, 1.2)	0.8726
	SNP	Case-CTRL	1 (0.6, 1.8)	0.9798	0.9 (0.7, 1.1)	0.1816	0.9 (0.7, 1.1)	0.2494
		Mother-CTRL	1.3 (0.7, 2.5)	0.4286	0.9 (0.7, 1.1)	0.2382	0.9 (0.8, 1.1)	0.4336
**Multiple CLP**	DIP Allele 1	Case-CTRL	1 (0.7, 1.4)	0.9697	1 (0.8, 1.2)	0.9333	1 (0.8, 1.2)	0.9358
		Mother-CTRL	0.8 (0.6, 1.2)	0.2857	0.9 (0.7, 1.2)	0.4737	0.9 (0.8, 1.1)	0.2955
	DIP Allele 2	Case-CTRL	1.1 (0.9, 1.4)	0.258	0.9 (0.5, 1.6)	0.6969	1.1 (0.9, 1.3)	0.4046
		Mother-CTRL	1.1 (0.9, 1.4)	0.2396	1.3 (0.8, 2.2)	0.3264	1.1 (0.9, 1.4)	0.1809
	DIP Allele 3	Case-CTRL	0.9 (0.7, 1.1)	0.323	1 (0.5, 2.2)	0.9053	0.9 (0.7, 1.1)	0.3998
		Mother-CTRL	1 (0.8, 1.3)	0.9303	0.7 (0.3, 1.7)	0.4316	1 (0.8, 1.2)	0.8883
	SNP	Case-CTRL	1 (0.5, 1.8)	0.9538	0.9 (0.7, 1.1)	0.2786	0.9 (0.8, 1.1)	0.3381
		Mother-CTRL	1.2 (0.6, 2.3)	0.5906	0.9 (0.7, 1.1)	0.2905	0.9 (0.8, 1.1)	0.4526
**Isolated CPO**	DIP Allele 1	Case-CTRL	1 (0.7, 1.4)	0.9498	1 (0.8, 1.2)	0.6933	1 (0.8, 1.2)	0.7915
		Mother-CTRL	0.9 (0.6, 1.2)	0.3981	0.8 (0.6, 1)	0.0708	0.9 (0.7, 1)	0.0808
	DIP Allele 2	Case-CTRL	1 (0.8, 1.3)	0.8703	0.5 (0.2, 1.1)	0.0749	1 (0.8, 1.2)	0.6497
		Mother-CTRL	1 (0.8, 1.3)	0.7614	1.1 (0.6, 1.9)	0.798	1 (0.8, 1.3)	0.7284
	DIP Allele 3	Case-CTRL	1.1 (0.9, 1.4)	0.3873	1.1 (0.5, 2.4)	0.7843	1.1 (0.9, 1.4)	0.3963
		Mother-CTRL	1.3 (1, 1.7)	**0.0431**^**3**^	1.1 (0.5, 2.4)	0.7368	1.2 (1, 1.5)	0.0576
	SNP	Case-CTRL	0.8 (0.4, 1.4)	0.4111	1 (0.8, 1.3)	0.7454	1 (0.8, 1.2)	1
		Mother-CTRL	0.8 (0.4, 1.5)	0.4739	1 (0.8, 1.3)	0.8894	1 (0.8, 1.2)	0.7186
**Multiple CPO**	DIP Allele 1	Case-CTRL	1 (0.7, 1.5)	0.9335	1 (0.8, 1.3)	0.9081	1 (0.8, 1.2)	0.8998
		Mother-CTRL	0.8 (0.6, 1.2)	0.3735	0.8 (0.7, 1.1)	0.2065	0.9 (0.7, 1.1)	0.1723
	DIP Allele 2	Case-CTRL	1 (0.8, 1.3)	0.8253	0.5 (0.2, 1.2)	0.1285	1 (0.8, 1.2)	0.7474
		Mother-CTRL	1 (0.8, 1.3)	0.824	1 (0.5, 1.9)	0.9076	1 (0.8, 1.3)	0.882
	DIP Allele 3	Case-CTRL	1.1 (0.8, 1.4)	0.5704	1 (0.4, 2.5)	0.9392	1.1 (0.8, 1.4)	0.598
		Mother-CTRL	1.2 (0.9, 1.6)	0.1179	1.4 (0.6, 3.1)	0.4179	1.2 (1, 1.6)	0.1044
	SNP	Case-CTRL	0.9 (0.5, 1.7)	0.7393	1 (0.8, 1.3)	0.9235	1 (0.8, 1.2)	0.847
		Mother-CTRL	0.7 (0.4, 1.4)	0.3247	1 (0.8, 1.3)	0.9406	1 (0.8, 1.2)	0.6925

^1^ OR = odds ratio

^2^ CI = confidence interval

^3^ Significant values are marked in bold.

## Conclusion

Our analysis shows no strong association between specific polymorphisms within the *MTHFD1L* gene and risk of cleft in an Irish cohort. The main limitation of our study would be sample size and the uncorrected p-values do indicate a possible association between the rs3832406 DIP and risk of CLP. However, we suggest further screening of rs3832406 DIP in a larger cohort and describe a new assay that will facilitate this. We have demonstrated that the modified Melting Curve Analysis developed for DIP rs3832406 could be a valid alternative to capillary electrophoresis for the genotyping of multiple allele deletion/insertion polymorphisms and can be employed by any laboratory with a Real-Time PCR instrument with melting curve capacity.

## Abbreviations

CLP, Cleft lip with or without cleft palate; CPO, Cleft palate only; DIP, Deletion/insertion polymorphism; HWE, Hardy-Weinberg equilibrium; LD, Linkage disequilibrium; MTHFD1, Methylenetetrahydrofolate dehydrogenase (NADP + dependent); MTHFD1L, Methylenetetrahydrofolate dehydrogenase (NADP + dependent) 1-like; MTHFR, Methylenetetrahydrofolate reductase; MTR, Methionine synthase; MTHFS, Methenyltetrahydrofolate synthetase; NTD, Neural tube defect; SNP, Single nucleotide polymorphism; TDT, Transmission disequilibrium test.

## Competing interest

The authors declare that they have no competing interests.

## Authors’ contributions

SM designed and carried out the genotyping assays, performed data analysis and drafted the paper. AM participated in the study design and execution, and commented on the manuscript. PK participated in the study design. JM participated in the study design and commented on the manuscript. JS participated in the study design. JT performed the statistical analyses. FP participated in the study design and commented on the manuscript. LB participated in the study design. APM participated in genotyping assay and study design, data interpretation and drafting of the paper. All authors read and approved the final manuscript.

## Pre-publication history

The pre-publication history for this paper can be accessed here:

http://www.biomedcentral.com/1471-2350/13/29/prepub
